# Defining and reporting treatment dropout in blended therapy for mental health: scoping review and analysis

**DOI:** 10.1038/s41746-026-02546-0

**Published:** 2026-03-17

**Authors:** Sophie Christine Eicher, Friederike Fenski, Solveig Behr, Leona Hammelrath, Johanna Boettcher, Carmen Schaeuffele, Christine Knaevelsrud

**Affiliations:** 1 Department of Education and Psychology, Freie Universität Berlin, Berlin, Germany; 2 Psychologische Hochschule Berlin, Berlin, Germany; 3 German Center for Mental Health (DZPG), partner site Berlin/Potsdam, Berlin, Germany

**Keywords:** Psychology, Health care

## Abstract

Evidence suggests that blended therapy combining face-to-face psychotherapy with digital components may reduce treatment dropout, yet definitions of dropout vary widely. This variability is particularly pronounced in blended therapy, where dropout may involve discontinuation of in-person sessions, disengagement from digital components, or both. This study aimed to identify operational definitions of treatment dropout in blended therapy and to examine how different definitions influence dropout rates, treatment outcomes, and usage patterns. A scoping review identified 14 studies reporting operational definitions of dropout. Five synthesized definitions were applied to data from a large blended therapy trial, revealing variation in dropout rates and their associations with depressive symptoms, anxiety, and life satisfaction. Cluster analysis further identified distinct digital usage patterns. These findings highlight the need for transparent and differentiated reporting of dropout definitions in blended therapy research to improve comparability and interpretation across studies.

## Introduction

Blended therapy is a promising treatment approach for mental disorders because it combines the strengths of face-to-face psychotherapy and (self-guided) digital health interventions: it offers the flexibility and accessibility of digital tools - such as structured online modules or app-based exercises that can be completed independently by patients - while still preserving the personal, responsive aspects of in-person sessions^1^. According to systematic reviews, blended therapy could potentially save clinicians time, support lasting changes from psychotherapy, and may even reduce treatment dropout rates^1,2^.

In face-to-face psychotherapy, dropout is defined as premature termination of the treatment, and usually operationalized as not attending all planned sessions^3^. Meta-analytic findings estimate dropout rates in face-to-face psychotherapy to range from 12% to 27%^4–8^. Already in 2005, Eysenbach emphasized the need for more specific research on dropout in digital health interventions, or so-called internet-based interventions (IBI), in his “law of attrition,” highlighting that disengagement is a common and complex phenomenon in such contexts^9^. This is also reflected in more variable definitions of dropout in the field of IBI: For example, “finished all available modules” or “completed number of recommended modules”, which could be less than the total numbers of modules^10^. Accordingly, dropout rates are highly variable, ranging from 15% to 65%^8,11–14^. Forbes et al.^10^ give a systematic overview of dropout operationalizations in IBI. Given the heterogeneous definitions applied, they conclude a need for a more standardized way to report treatment dropout metrics. Blended therapy, with combined face-to-face and digital components, adds another layer of complexity: dropout may involve discontinuing in-person sessions, disengaging from digital tools, or both. However, a closer examination of the included studies reveals significant inconsistencies in how dropout is operationalized. Some studies, such as Christensen et al.^15^, do not provide an explicit operationalization of dropout, while others, such as Campbell et al.^16^ and Kiluk et al.^17^, define treatment completion narrowly, based only on attendance of a minimum number of face-to-face sessions.

Blended therapy might help to reduce treatment dropout through more consistent engagement and guidance^18^, but the inconsistencies in definitions hinder the ability to interpret findings, compare results across studies, and draw generalizable conclusions about dropout rates and their implications for patient outcomes. This lack of standardization creates challenges for clinicians, who struggle to translate inconsistent findings into practical retention strategies, for researchers, who face difficulties in synthesizing evidence to advance the field, and it complicates decision-making for policymakers regarding the allocation of healthcare funding for specific interventions.

Treatment dropout in blended therapy might be associated with treatment outcomes. Patients who dropped out of face-to-face psychotherapy for depression showed poorer treatment outcomes in comparison with completers^19^. Evidence from guided IBI highlights the critical role of treatment completion in determining outcomes, too. For instance, Moshe et al.^20^ demonstrated a strong dose-response relationship between session completion and effect size, emphasizing that treatment completion significantly improves therapeutic outcomes. Wright et al.^21^ also reported that higher completion rates were consistently associated with better outcomes. These findings from face-to-face psychotherapy and digital interventions suggest that the relationship between treatment dropout and outcomes likely extends to blended therapy as well, but more research is needed. In blended therapy, disengagement from one component (e.g., the digital component) may not equate to total treatment dropout. However, dropping out of one component could still diminish the overall effectiveness of the treatment. Conversely, consistent engagement across digital and face-to-face components may amplify therapeutic benefits. Thus, understanding and standardizing dropout definitions in blended therapy is essential not only for accurately measuring dropout rates but also for identifying how varying levels of engagement across components influence patient outcomes.

To move beyond conceptual distinctions and empirically capture the level of engagement, indicators that reflect actual patient behavior are required. Although meta-analytic evidence is lacking, some studies indicate that adding usage data improves the prediction of treatment dropout in IBI when combined with other predictors (e.g., sociodemographic variables). Bremer et al.^22^ reported that the average number of days needed to complete each module of an IBI was a strong predictor for dropout. Moshe et al.^23^ showed that adding usage data measures, such as days taken to complete a module and minutes spent online to complete each module, were useful in providing a more accurate prediction of treatment dropout. While these findings derive from IBI, they underscore the potential of usage data to improve dropout predictions in blended therapy. So far, few studies have investigated usage patterns in blended therapy. Krijnen-de Bruin et al.^24^ distinguished between “low” and “regular” users in a case-by-case, personalized intervention for anxiety and depression. Kemmeren et al.^25^ identified three distinct usage groups, emphasizing “mainly web-based,” “mainly face-to-face,” and “blended compliant” users in an integrated, alternate and standardized intervention for depression. Wu et al.^26^ found that poor early digital engagement, defined as completion of all assigned digital activities prior to the second face-to-face session, was a strong predictor of treatment dropout in an integrated, alternate and personalized intervention for depression and anxiety. However, no studies have yet investigated how usage patterns over the course of the entire intervention are associated with treatment dropout. This is a critical gap, as identifying patterns linked to dropout could guide the design of interventions to enhance engagement and adherence. By exploring how different usage patterns are linked to dropout, we can identify at-risk patients and design more personalized interventions to prevent dropout and improve outcomes.

The absence of standardized definitions and the lack of research connecting dropout to outcomes and usage patterns with dropout in blended therapy present significant barriers to the efforts of designing better interventions. To address these gaps, our study attempts to provide a comprehensive understanding of treatment dropout in blended therapy. Specifically, we focus on two key aims: first, to conduct a scoping review to identify operational definitions of dropout in the blended therapy literature, and second, to investigate the identified operational definitions using real-world data on blended therapy. For the second aim, the key objectives are to compare dropout rates for identified definitions (RQ.1), to investigate the association between treatment dropout and mental health outcomes (RQ.2), and to explore how different usage patterns relate to dropout (RQ.3).

## Results

### Scoping review of operational treatment dropout definitions (aim 1)

**Fig. 1:**
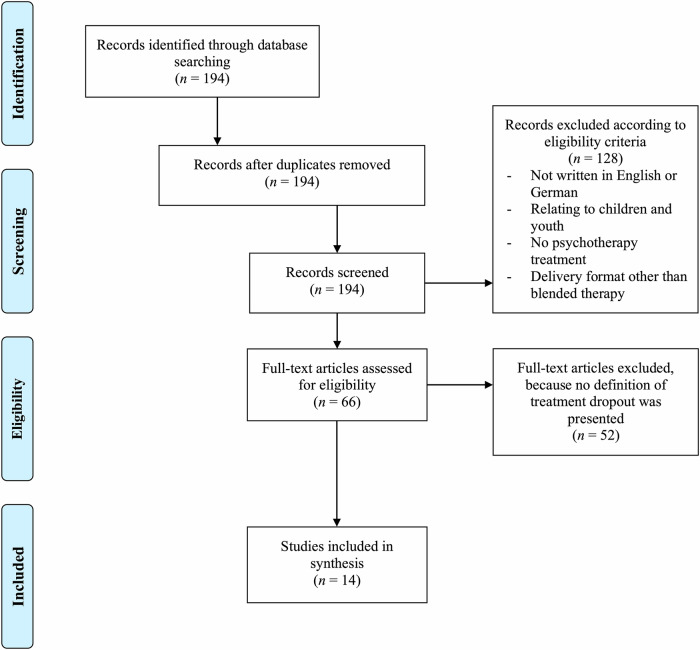
PRISMA flowchart for the scoping review. The flowchart illustrates the process of identification, screening and including studies for the scoping review. *N* represents the number of studies.

Figure 1 shows the detailed screening process for the scoping review. The literature search identified *N* = 194 publications. After screening, a total of *n* = 14 publications provided an operational definition of treatment dropout and were included in the scoping review. Table 1 gives an overview of the operational definitions identified in the scoping review.

**Table 1:** Operational definitions identified in the scoping review

Authors	Operational definition
Only digital component	
Birnkammer and Calvano^27^	Completion is defined as starting the last module, e.g., interacting with 100% of the modules
Only face-to-face component	
Branquinho et al.^28^	Number of completed face-to-face sessions, less than 75% was categorized as dropout
Nordby et al.^29^	Number of completed face-to-face sessions, less than 75% was categorized as dropout
Pérez et al.^30^	Participants who discontinued intervention because of the request to leave the study or changing the face-to-face provider were categorized as dropout
Wu et al.^26^	Participants who completed not more than one face-to-face session were categorized as dropout
Combination (additive)	
Breider et al.^31^	Number of completed digital and face-to-face session, less than 100% was categorized as dropout
Forrer et al.^32^	Number of completed digital and face-to-face session, less than 100% was categorized as dropout
Kooistra et al.^34^	Number of completed digital and face-to-face session, less than 100% was categorized as dropout
Lundin et al.^35^	Number of completed digital and face-to-face session, less than 50% was categorized as dropout
Mathiasen et al.^36^	Number of completed digital and face-to-face session, less than 75% was categorized as dropout
Romijn et al.^37^	Number of completed digital and face-to-face session, less than 100% was categorized as dropout
Combination (complex)	
Garety et al.^33^	Adherence was operationalized as at least 1 home screen interaction after a minimum of 3 therapy sessions
Combination (imprecise/not applicable)	
Fernández-Buendía^39^	Participants who explicitly declare to not wanting to continue with treatment, or who do not respond to reminders will be categorized as dropout
Schuster et al.^38^	Adherence was operationalized as the percentage of completed face-to-face sessions

### Synthesis of literature search results

Supplementary table 1 provides a detailed overview of all included studies. *N* = 1 study included only the digital component for operationalizing treatment dropout^27^, *n* = 4 studies included only the face-to-face component^26,28–30^, and *n* = 7 studies combined the digital and face-to-face component for their operational definition of dropout^31–37^. *N* = 2 studies were not clearly categorizable^38,39^ (included digital and face-to-face component but do not report if and how it is combined). In summary, three categories of definitions emerged: definitions that rely solely on the face-to-face component, definitions that rely solely on the digital component, and definitions that rely on some sort of combination of the two components (see Table 1 for a detailed overview). Most definitions are based on the percentage of content (digital, face-to-face, or both) completed, with criteria ranging from 50% to 100% of content completion. Only one definition comprised a more complex combination of components were “adherence was operationalized as at least 1 home screen interaction after a minimum of three therapy session^33^.”

Building on the classification by Ferrao Nunes-Zlotkowski et al.^1^, most studies can be categorized as employing an integrated interaction between digital and face-to-face components, typically following an alternating delivery pattern with standardized content assignment (see Supplement). Notably, only one study reported the use of personalized content assignment^26^. Regarding the role of the components in the intervention, only Kooistra et al.^34^ identified the face-to-face component as supplementary to the digital *core* component. In contrast, the other studies did not explicitly designate either component as core or supplementary.

### Application of identified definitions to real-world data (aim 2)

The operational definitions of treatment dropout identified in the literature vary in scope and focus, including either only digital or face-to-face components, or a combination of both. Some of the identified operational definitions appear to rely heavily on the specific context of the blended therapy intervention. After synthesizing existing definitions from the scoping review, we applied five operational definitions to the PsyTOM dataset (see Table 2). The criteria for face-to-face components used in other studies, which are the amount of prescribed and then attended sessions, cannot be directly applied to the PsyTOM study. The PsyTOM study was conducted in a routine care setting; hence, there was no predetermined number of face-to-face sessions as part of the trial. Consequently, data on the number of intended face-to-face sessions were not available. Therefore, we relied on the therapist’s evaluation of treatment dropout (Was the therapy ended earlier than originally planned? (Yes/No)) to determine dropout in the face-to-face component and could not include information about the percentage of face-to-face sessions completed. For the digital component, we used a variable that tracked the percentage of assigned digital components completed by each participant.

**Table 2:** Identified definitions and operationalizations with PsyTOM data

	Identified definition	Operationalization with PsyTOM data
Operational definition 1 (therapist evaluation)	Participants who did not complete 75% or 100% of the face-to-face component are considered dropouts	Participants who were considered dropouts by their therapist are considered dropouts^1^
Operational definition 2 (digital-only)	Participants who did not complete 100% of the digital component are considered dropouts	Participants who did not complete 100% of assigned digital components are considered dropouts
Operational definition 3 (100% digital & therapist)	Participants who did not complete 100% of the digital component and face-to-face component are considered dropouts	Participants who did not complete 100% of the digital components and who were considered dropouts by their therapist are considered dropouts
Operational definition 4 (75% digital & therapist)	Participants who did not complete 75% of the digital component and face-to-face component are considered dropouts	Participants who did not complete 75% of the digital components and who were considered dropouts by their therapist are considered dropouts
Operational definition 5 (50% digital & therapist)	Participants who did not complete 50% of the digital component and face-to-face component are considered dropouts	Participants who did not complete 50% of the digital components and who were considered dropouts by their therapist are considered dropouts

^1^Based on the therapist-rated item. Was the therapy ended earlier than originally planned? (Yes/No).

For all the following analyses, we used the PsyTOM trial data of participants in the blended therapy arm who either completed or dropped out of therapy during the study period (*n* = 147). The sample was mostly female, rather young and mostly well educated (see Table 3).

**Table 3:** Sample characteristics

Variables			
Age in years (*M,* SD, Range*)*		*M* = 35.0; SD = 12.6	18–72
Gender (*n*, %)			
	Female	97	66.0%
	Non-binary	1	0.7%
	Gender fluid	1	0.7%
	Male	47	32.0%
	Other	0	0.0%
	No information	1	0.7%
Education (*n*, %)			
	No degree	0	0.0%
	Nine years of education	8	5.4%
	Secondary school certificate	21	14.3%
	College entrance qualification	81	55.1%
	Vocational education	36	24.5%
	Other	1	0.7%
PHQ-8 (T0) (*M,* SD, Range*)*		*M* = 12.3; SD = 5.3	2–24
GAD-7 (T0) (*M,* SD, Range*)*		*M* = 10.8; SD = 4.9	0–21
SWLS (T0) (*M,* SD, Range*)*		*M* = 18.7; SD = 6.0	5–34

### Consistency in dropout rates of different operational definitions of treatment dropout (RQ 1)

The dropout rate varied based on the definition applied (Table 4). For example, Definition 2 - completion of 100% of assigned digital content—produced a very high dropout rate (93.8%), while Definition 1—therapist-rated dropout—resulted in a much lower rate (67.3%). Definitions 1, 3, 4, and 5, which incorporate therapists’ evaluations, classified fewer participants as dropouts compared to Definition 2 (digital-only), which categorized most participants as dropouts. Although Definitions 3 (100% digital & therapist) and 4 (75% digital & therapist) identified a similar number of dropouts to Definition 1, they only applied to a subset of participants and resulted in missing values for about a third of the participants reflecting gaps in the operational criteria based on the definitions from the scoping review (for example, the combination “completed 100% of digital content” and “therapist evaluated participant as dropout” was not accounted for in the operational definition and thus resulted in a missing value.). Definition 5 (50% digital & therapist) classified the least participants as dropouts, but simultaneously, produced the highest number of missing values.

**Table 4:** Treatment dropout frequencies and rates for each definition

	*N* = 147			%		
	Dropout	Completer	NA	Dropout	Completer	NA
Definition 1 (therapist evaluation)	99	48	0	67.3	32.7	
Definition 2 (digital-only)	135	9	3	91.8	6.1	2.0
Definition 3 (100% digital & therapist)	91	4	52	61.9	2.7	35.4
Definition 4 (75% digital & therapist)	82	9	56	55.8	6.1	38.1
Definition 5 (50% digital & therapist)	71	17	69	48.3	11.6	40.1

### Association of treatment dropout with treatment outcomes (RQ 2)

For definition 4 (75% digital & therapist) and definition 5 (50% digital & therapist), almost all group differences were significant, with participants who dropped out reporting higher scores for depressive symptoms and anxiety symptoms and lower scores of satisfaction with life at baseline and 6-month follow-up (see Table 5). For definition 1 (therapist-only), definition 2 (digital-only), and definition 3 (100% digital & therapist), there were no significant group differences for all scales and timepoints (see Table 5). In conclusion, the results show that significant group differences between dropouts and non-dropouts on certain measures are dependent on the definition of dropout applied, even though all other definitions showed descriptive trends (see Table 5). These results should be interpreted with caution, as group sizes varied across definitions.

**Table 5:** Associations between treatment dropout and depressive symptoms, anxiety symptoms and satisfaction with life

		Definition 1 (therapist-only)			Definition 2 (digital-only)		
		Dropout (mean, SD)	Completer (mean, SD)	MPV	Dropout (mean, SD)	Completer (mean, SD)	MPV
PHQ	T0	12.8, 5.4	11.2, 4.9	0.867	12.5, 5.2	8.9, 4.8	0.415
	T3	9.1, 4.0	7.8, 3.9	0.478	8.7, 4.0	7.6, 4.2	1
GAD	T0	11.5, 4.9	9.2, 4.4	0.080	10.9, 4.9	8.0, 3.7	0.669
	T3	7.9, 3.8	6.3, 3.6	0.237	7.4, 3.8	6.6, 3.7	1
SLWS	T0	17.5, 5.7	21.0, 5.8	0.053	18.7, 6.1	20.7, 4.8	1
	T3	20.3, 4.8	21.7, 6.3	1	20.7, 5.4	22.8, 5.3	1

		Definition 3 (100% digital & therapist)			Definition 4 (75% digital & therapist)			Definition 5 (50% digital & therapist)		
		Dropout (mean, SD)	Completer (mean, SD)	MPV	Dropout (mean, SD)	Completer (mean, SD)	MPV	Dropout (mean, SD)	Completer (mean, SD)	MPV
PHQ	T0	13.0, 5.4	8.5, 4.4	0.778	12.7, 5.2	7.2, 3.6	0.022*	12.8, 5.2	9.2, 4.7	0.082
	T3	9.1, 4.0	4.8, 3.6	0.416	9.0, 3.8	5.0, 2.7	0.019*	9.1, 3.9	5.3, 3.2	0.004**
GAD	T0	11.6, 5.0	6.5, 2.6	0.375	11.3, 4.9	6.3, 2.3	0.027*	11.6, 4.9	7.4, 3.9	0.018*
	T3	7.8, 3.8	4.3, 2.6	0.535	7.8, 3.7	3.8, 2.3	0.014*	8.1, 3.6	3.9, 2.9	<0.001***
SLWS	T0	17.5, 5.8	20.3, 4.9	1	17.3, 5.8	24.6, 6.0	0.048*	17.2, 6.0	24.6, 5.6	0.002**
	T3	20.3, 4.8	24.5, 5.9	1	20.2, 4.6	25.4, 5.1	0.088	20.4, 4.7	24.8, 6.8	0.034*

*MPV* pooled median *p*-value.

* for *p* < 0.05, ** for *p* < 0.01, and *** for *p* < 0.001.

### Usage patterns of the digital component (RQ 3.1)

We used an elbow plot and silhouette scores to determine the optimal number of clusters for the clustering algorithm. The elbow plot initially suggested four clusters after 10 and 15 iterations. However, a five-cluster solution provided clearer and more interpretable patterns in the usage data (descriptives see Table 6), leading us to select this as the best fit. The silhouette score for the five-cluster solution indicated a moderate model fit (mean = 0.24) and alternative cluster numbers did not yield higher silhouette scores.

- Cluster 1 (therapist contact) represented users with the highest amount of therapist interactions (*n* = 16), indicating stronger reliance on therapist support. This group had more assigned chapters and was characterized by above-average engagement and high completion rates.
- Cluster 2 (selective usage) represented users with a below-average number of assigned chapters, moderate engagement and minimal therapist interaction (*n* = 12). However, participants in this cluster had the longest average session duration.
- Cluster 3 (minimal usage) represented users with the lowest engagement across all metrics. Participants had a below-average of assigned chapters but showed low completion rates and low additional module requests (*n* = 52).
- Cluster 4 (additional content) represented high module requests but average session metrics and little therapist interaction (*n* = 24). This group exhibited average login frequency and duration, but they stood out for requesting the most additional modules, suggesting a preference for extra content.
- Cluster 5 (moderate usage) represents average usage (*n* = 40). Participants in this cluster showed moderate engagement across all metrics, but high completion rates. They had minimal therapist interactions and requested few additional modules.

**Table 6:** Descriptives usage data and z-transformed values for cluster centroids

Variable	Entire sample		Cluster 1 (therapist contact)	Cluster 2 (selective users)	Cluster 3 (minimal users)	Cluster 4 (additional content)	Cluster 5 (moderate users)
	Mean (SD)	Range					
Number of logins	15.5 (14.1)	1–92	2.14	−0.72	−0.58	−0.02	0.13
Total duration of logins (minutes)	276.2 (220.2)	24–1312	1.91	−0.44	−0.70	−0.05	0.30
Mean duration of logins (minutes)	19.5 (6.4)	9–46	-0.62	2.35	−0.34	−0.20	0.11
Number of assigned chapters	5.6 (5.6)	0–36	1.86	−0.46	−0.61	−0.05	0.22
Number of requested modules	1.3 (2.0)	0–10	0.34	−0.26	−0.51	1.71	−0.42
Number of messages from therapist to patient	0.1 (0.4)	0–2	1.10	−0.24	−0.04	−0.24	−0.18
Number of messages from patient to therapist	0.1 (0.6)	0–6	0.94	−0.13	−0.10	−0.13	−0.13
Assigned components completed (percentage)	0.3 (0.3)	0–100	0.77	0.07	−0.80	−0.31	0.88

### Impact of dropout definition (RQ3.2 and RQ3.3)

Across all clusters, the identification of treatment dropout cases was consistent across all definitions (see Table 7): in every cluster, more participants were categorized as dropouts than non-dropouts, regardless of the dropout definition. However, in comparison to Definition 1, the other definitions that relied more heavily on the digital component consistently classified more participants as dropouts in each cluster. The operational definitions applied classified almost all participants as dropouts, even if they showed moderate or high engagement metrics (Tables 6 and 7). Across all definitions, dropout was lowest in Cluster 1 and highest in Cluster 3.

**Table 7:** Usage behavior and dropout frequencies

	Definition 1 (therapist evaluation)		Definition 2 (digital-only)			Definition 3 (100% digital & therapist)			Definition 4 (75% digital & therapist)			Definition 5 (50% digital & therapist)		
	Dropout	Completer	Dropout	Completer	NA	Dropout	Completer	NA	Dropout	Completer	NA	Dropout	Completer	NA
Cluster 1 (therapist contact)	6	10	15	1	0	5	0	11	3	2	11	1	4	11
Cluster 2 (selective users)	9	3	10	2	0	8	1	3	8	1	3	7	2	3
Cluster 3 (minimal users)	37	15	52	0	0	37	0	15	37	0	15	37	0	15
Cluster 4 (additional content)	19	5	24	0	0	19	0	5	18	0	6	17	2	5
Cluster 5 (moderate users)	25	15	34	6	0	22	3	15	16	6	18	9	9	22

## Discussion

This is the first study to investigate the impact of different operationalizations of treatment dropout in blended therapy. We first identified operational definitions in the literature and then compared them using real-world data.

We conducted a scoping review to identify existing definitions of treatment dropout in blended therapy. Some studies considered only the digital or face-to-face component for their operational definitions, while others used various combinations of both. By applying these definitions to our trial data, we found that, first, some definitions from the literature were too tailored to their specific study designs to be applied to our data, and second, treatment dropout rates in our data changed depending on the definition applied. The scoping review revealed that operational definitions depend very much on the context and overall structure of a blended therapy intervention. For some interventions, face-to-face sessions might be the core component, and the digital component supplementary. For other interventions it might be the other way around with the digital component as core component. For integrative set-ups, the different face-to-face and digital modules might build on each other, resulting on a higher importance of completing both components, especially if they follow an alternate structure. Moreover, assignment of digital components can be standardized or personalized, potentially resulting in different criteria for treatment completion^1^. The range of operational definitions identified in the literature search reflects this variability in blended therapy intervention setups. This heterogeneity complicates direct comparisons, highlighting the importance of reporting results in a manner that facilitates comparability across different studies. One recommendation for researchers is to at least report separate dropout rates for the digital and face-to-face components in addition to the overall rate derived from the study-specific definition of treatment dropout.

We used data from the PsyTOM study to compare dropout definitions, acknowledging that this data is also influenced by its contextual setting. PsyTOM is integrated in routine care, meaning participants are already in an outpatient therapy setting, so naturally, there is an emphasis on the face-to-face component and the contact with the therapist. This might explain why the results highlight the importance of the therapist’s evaluation in determining dropout status and why only relying on the digital component resulted in substantially higher dropout rates. This was demonstrated in differences in dropout rates by about 25% between only looking at digital module completion and the therapist’s evaluation only. The other definitions that included the therapist evaluation and the digital content completion yielded dropout rates closer to the therapist evaluation-only definition, suggesting that solely relying on digital engagement metrics may not be the best indicator of treatment dropout in this type of blended therapy. The fact that therapist-evaluated dropouts resulted in lower dropout rates than digital module completion alone indicates that therapists may consider additional factors when classifying someone as a dropout - perhaps especially in settings where face-to-face therapy is the core component of blended therapy setups. For example, therapists might assess patient engagement through interactions during face-to-face sessions and by evaluating the need for treatment adaptability to individual circumstances, aspects that cannot be captured by digital metrics alone. This perspective also aligns with core findings from psychotherapy research: factors such as the therapeutic alliance and the quality of interaction between patient and therapist are consistently predictors of treatment success^40^. In IBI, guidance has likewise been shown to significantly enhance adherence and outcomes^41,42^. It may therefore not be the amount of digital content completed that determines treatment success or continuation, but how well the digital elements are integrated into a therapeutic context that is supportive and responsive to the individual needs of the patient. This view aligns with research showing that both therapists and patients value the ability to tailor blended therapy protocols to personal circumstances and preferences^43,44^. Moreover, personal circumstances, such as time constraints due to other responsibilities or engagement with therapy content outside of digital modules, are difficult to assess when only relying on digital usage data or the number of intended face-to-face sessions. This suggests that in some settings, therapist and patient input might be crucial for understanding true disengagement and dropout risk in blended therapy. However, digital engagement can also play a crucial role, depending on the setting. Wu et al.^26^ found that poor digital engagement was associated with a higher likelihood of treatment dropout—specifically, clients who did not complete the digital activities assigned by their provider early in treatment were significantly more likely to drop out. This suggests that while therapist evaluations may provide a more comprehensive view of patient treatment engagement, early digital engagement may still be an important predictor of dropout risk, particularly in blended therapy interventions where digital components are more central. In the blended therapy model used in the PsyTOM study, the face-to-face component serves as the primary treatment, while digital modules function as supplementary exercises. Many participants did not complete all digital modules, but were not evaluated as dropouts by their therapists. While this finding may be relevant to other blended therapy interventions, its applicability likely depends on the relative emphasis placed on digital vs. face-to-face components. Future research should further explore how therapist evaluations and digital engagement metrics interact to provide a more comprehensive understanding of treatment dropout risk in blended therapy.

In addition to differences in dropout rates, we examined how different operational definitions of dropout influenced associations with key mental health outcomes, including depressive symptoms, anxiety, and satisfaction with life. Compared with completers, participants who had dropped out reported worse mental health outcomes post-treatment at least descriptively. This is in line with prior research that showed that treatment completion is associated with better outcomes^20,21^. Under definitions 4 (75% digital & therapist) and 5 (50% digital & therapist), there were significant differences in anxiety, depressive symptoms, and satisfaction with life between dropouts and completers, and all other definitions showed descriptive trends. These findings suggest that the association between treatment dropout and mental health outcomes remains consistent, regardless of the dropout definition applied. Higher baseline symptom severity and lower quality of life are associated with increased dropout rates^23,45^. Our findings align with this pattern: Across all dropout definitions, participants who dropped out had higher levels of depressive and anxiety symptoms and lower satisfaction with life at baseline compared to completers. These differences were primarily descriptive for definitions 1 (therapist evaluation), 2 (digital only), and 3 (100% digital & therapist) but reached statistical significance under definitions 4 and 5. Thus, participants with higher initial symptom burden may have been at greater risk of disengaging from treatment. This reinforces the importance of early identification of at-risk individuals to enhance retention in blended therapy interventions. The inconsistencies in associations with outcomes across dropout definitions present a challenge for ensuring the validity and generalizability of findings in blended therapy research. Depending on how dropout is defined, the strength of associations with treatment outcomes may vary, leading to differing interpretations of dropout’s impact on symptom change. One potential explanation is that participants who ultimately drop out may experience lower early treatment benefits, contributing to disengagement^46^. However, alternative explanations, such as external life factors or low initial motivation, should also be considered^47–49^. To better understand dropout dynamics in blended therapy, future research should adopt both quantitative and qualitative approaches. While meta-analyses can summarize research results on early engagement patterns and symptom trajectories as predictors of dropout risk, more qualitative studies are needed to explore patient- and therapist-reported reasons for disengagement. Existing work, such as that of Wilhelmsen et al.^50^ and Wells et al.^51^, has provided valuable insights into patient-reported reasons for dropout, for example, time constraints and attitudes towards treatment. However, studies that integrate both patient and therapist perspectives are still lacking. A next step in addressing this gap is to integrate both perspectives (see preregistration, 10.17605/OSF.IO/G84K9) to understand the different processes that lead to disengagement from blended therapy.

After identifying various usage patterns for the digital component in the PsyTOM data, our results show some variation in dropout classification based on usage patterns across different definitions. Definitions that relied more strongly on the digital component tended to classify a larger number of participants as dropouts, even among those with moderate or high usage levels, which suggests that relying on usage data alone may again overestimate dropout risk in our data. This points to a broader research question in the context of digital and blended therapy, that goes beyond the concept of treatment dropout: How can adherence to the intervention be effectively measured? The concept of adherence refers to “following the prescribed recommendations^52^.” In the context of digital interventions, Donkin et al.^53^ describe adherence as “the degree to which the user followed the program as it was designed”, aligning with the concept of intended use. As of right now, there are still many questions surrounding adherence: one important issue in this regard is understanding the right dosage in blended therapy, in order to establish a criterium for intended use. Sieverink et al.^54^ documented in a systematic review that only six studies reported a justified operationalization for the intended use of an intervention. Though most studies operationalized adherence as “the more use, the better”^54^. This idea seems to play into some operational definitions of treatment dropout, too, classifying participants as dropouts if they don’t complete 100% of the provided content^31,32,37^. However, the justification for this specific percentage remains unanswered. As described, dropout may involve qualitative differences, such as symptom improvement or the achievement of treatment goals^55^. However, these aspects are typically disregarded when applying criteria such as completing “100% of the provided content”. In their systematic review, Ferrao Nunes-Zlotkowski et al.^1^ identified the “good enough effect” as a barrier to intervention engagement in blended therapy. This phenomenon describes patients discontinuing therapy because they feel better or believe further intervention is unnecessary^1,55^. Therefore, not all dropouts are inherently negative. Especially in digital components, where flexibility and autonomy are higher, disengagement may reflect informed choices rather than failure. This underscores the importance of future research that examines not only when and how dropout occurs, but also why, ideally by incorporating patient-centered approaches. Additionally, a more fine-grained reporting is needed, including the timing of dropout within the intervention and concurrent symptom trajectories, to better understand the diverse reasons behind disengagement.

The application of the results of the scoping review to our real-world data revealed varying dropout rates, highlighting the importance of reporting consistent and comparable definitions across studies. Due to heterogeneity in blended therapy interventions, it might be difficult to recommend a universally applicable definition for all studies. However, our findings indicate that both digital and face-to-face components should be considered, and therapist evaluations included when reporting treatment dropout. To improve comparability and interpretability across studies, we recommend reporting dropout rates separately for digital and face-to-face components, along with the operational definition applied in the study context. Additionally, specifying the blended therapy classification according to Ferrao Nunes-Zlotkowski et al.^1^ and including the intended use of intervention components would improve transparency. Reporting the expected level of engagement and including dropout timing with symptom tracking would help differentiate between treatment dropout and termination of treatment due to symptom improvement. Adding this information would provide a more nuanced understanding of treatment dropout in a specific study context and facilitate comparisons across studies, even if the blended therapy setup differs. Looking ahead, as the boundaries between digital and face-to-face psychotherapy become increasingly fluid and interactive and will potentially be shaped by AI-supported components, static, percentage-based definitions of dropout may no longer be sufficient. We see this work as a starting point to encourage more flexible and context-sensitive operationalizations of dropout in future research.

This study highlights the significant heterogeneity between different blended therapy setups, such as alternate versus case-by-case and integrated versus sequential approaches or those focusing primarily on face-to-face versus digital components. While comparisons across these setups could provide more nuanced insights into dropout differences, this was beyond the scope of the present study. One limitation of this study is the incomplete dataset regarding information about the dropout status of the participants. At the time of the end of data collection, most participants (approximately 70%) had not yet completed their treatment, and only data from participants who had either completed or dropped out were included. Of this subset, therapists reported a higher proportion of dropouts compared to completers. This is why, compared to dropout rates found for face-to-face psychotherapy (16%–30%) and internet-based interventions (15%–65%), the dropout rates for the different operationalizations in our study appear quite high (48%–92%). Therefore, this dataset is not suitable for such a comparison of dropout rates between different settings. Given the large amount of missing data, imputation for treatment dropout was not feasible. Additionally, we had no information about the number of intended face-to-face sessions. As a reference for treatment dropout, we had to rely on the therapist-rated item, Was the therapy ended earlier than originally planned? (Yes/No). Although this was the only available indicator for dropout, it does not explicitly distinguish between treatment dropout and early termination due to the patient reaching treatment goals earlier than expected. Future research could benefit from more detailed measures to better capture these distinctions. Furthermore, the model fit for the usage data clusters was suboptimal, suggesting that engagement in blended therapy is more complex than the variables used in this study could capture. While the usage variables included were informed by existing literature^23–25^, and like other authors^24,56^ we found groups of minimal users and moderate users, future studies could benefit from exploring advanced methodologies, such as machine learning, to identify more relevant variables for informing usage patterns in blended therapy interventions. Lastly, the change in analysis method from survival analysis to logistic regression limits the interpretability of our findings with respect to our preregistered research question. In particular, we are unable to make statements about the timing of dropout, only about its association with usage patterns. Moreover, the interpretation of the logistic regression results is limited, as several models showed signs of separation and numerical instability. Overall, both the cluster analysis and the subsequent logistic regressions are exploratory in nature and should be interpreted with caution.

Treatment dropout definitions in blended therapy are heterogeneous. Including the therapist evaluation may offer a valuable perspective beyond considering dropout from the face-to-face or the digital components in blended therapy in isolation, especially if the digital components are integrated into a routine care setting. To draw comparisons with other studies, we recommend reporting the specific operational dropout definition applied in the study context, as well as separate dropout rates for digital and face-to-face components. We further recommend including a classification of the blended therapy approach, as well as information about the intended use and dropout timing when reporting treatment dropout rates for blended therapy interventions.

## Methods

### Scoping review

For aim 1, we conducted a literature review, following the methodology of a scoping review^57^ (PRISMA-ScR) to understand how treatment dropout in blended therapy for mental health is defined in the literature. This approach clarifies and identifies key concepts or definitions (here: treatment dropout) related to a concept (here: blended therapy) based on studies identified through a systematic literature search^58^. The literature search was conducted in January 2025 in PubMed with the search string: (“blended”) AND (“mental” OR “psychological” OR “psychotherapy”) AND (“dropout” OR “adherence” OR “attrition” OR “termination” OR “engagement” OR “usage”). Following the PCC criteria^57^ (participants, concept, context), two individuals screened the results separately. We included primary research (including study protocols) on individuals receiving psychotherapy (participants) that was delivered in a blended treatment approach (*context*, digital component and face-to-face component) and that reported treatment dropout (concept*)*.

### Practical application of derived dropout operationalizations with real-world data

We then applied the derived operationalization (aim 1) to the data from the PsyTOM trial (aim 2).

The PsyTOM trial, registered in the German Clinical Trials Register (DRKS00028536) on 07.06.2022, investigated the effectiveness of blended therapy (BT) compared to treatment as usual (TAU) in a naturalistic routine care setting in Germany. In BT, psychotherapists could use TONI, a comprehensive, transdiagnostic, and transtheoretical internet-based toolkit composed of modular therapeutic content. Psychotherapists working in routine outpatient care under public health insurance were eligible to participate if they had internet access in their practice. Patients were enrolled in the study by their therapists based on the following inclusion criteria: age over 18, sufficient German language proficiency, internet access, and the ability to read and write. All participants received routine psychotherapy provided by licensed psychotherapists. Treatment lengths and procedures varied in accordance with the naturalistic design, and no restrictions were placed on additional healthcare utilization. A more detailed description of the study design is available in Schaeuffele et al.^59^.

In the (BT) condition, psychotherapists could flexibly integrate TONI into routine psychotherapy. TONI is a digital toolkit comprising twelve transdiagnostic, transtheoretical modules (see Supplement), as well as self-monitoring tools. Therapists could individualize treatment by selecting specific modules, chapters, and usage formats, without predefined instructions regarding frequency, duration, or implementation. Patients could request access to modules, and therapists had the option to view module inputs, enable messaging, and incorporate other TONI components, such as symptom monitoring, into the therapeutic process. Additionally, participants and therapists could exchange messages. A more detailed description of the intervention is available in the corresponding publications^59,60^.

### Sample

We investigated the different operationalization in the BT arm of the PsyTOM trial RCT (*n* = 583). Due to the naturalistic nature of the trial and the absence of a fixed treatment duration, many patients in the BT arm were still receiving psychotherapy at the end of the data collection period. Thus, we included only those BT participants who had either completed or dropped out of treatment during the study period (*n* = 147). The hypotheses and planned analyses were preregistered on the Open Science Framework (10.17605/OSF.IO/G84K9).

### Primary outcome

The primary outcome was treatment dropout. As we aimed to compare definitions, we conducted all analyses using different operationalizations of dropout as identified through the scoping review. As a reference, we used the therapist-rated item from the PsyTOM study, Was the therapy ended earlier than originally planned? (Yes/No).

### Covariates

Depressive symptoms were assessed with the 8-item German version of the Patient Health Questionnaire^61–63^ (PHQ-8). Anxiety symptoms were assessed with the 7-item German version of the Generalized Anxiety Disorder Scale-7^64,65^ (GAD-7). Satisfaction with life was assessed with the 5-item German version of the Satisfaction with Life Scale^66,67^ (SWLS). Participants completed questionnaires at baseline (t0), after 6 weeks (t1), after 12 weeks (t2) and after 6 months (t3). For the analysis, we will use the assessment at t0 and t3.

### Statistical analysis

We performed all analyses using RStudio (Version 2023.9.0.463). During the pre-processing of the data, missing data for mental health outcomes were handled by using multiple imputation. For a full overview of data preparation, see Schaeuffele et al.^68^ and the corresponding OSF project (10.17605/OSF.IO/PRE87). The code for all following analyses is provided on OSF (https://osf.io/fh7ku/?view_only=b17783ddb00d4f4f9cd1e1c351d92d67).

To address RQ1 (compare dropout rates for identified definitions), we used descriptive analyses of treatment dropout rates. We calculated treatment dropout rates (absolute and relative frequencies) for therapists’ evaluation of dropout, and by applying the operational definitions identified from the literature review. Due to violation of the normality assumption, we relied on Wilcoxon-Rank-Test to evaluate differences in depressive symptoms, anxiety symptoms, and satisfaction with life between participants who dropped out and those who did not (RQ.2). As the Wilcoxon test is not directly supported by Rubin’s rules for pooling results across multiple imputations, we followed the recommendation by Eekhout et al.^69^ and calculated the median of the *p*-values obtained from each of the 30 imputed datasets as an overall pooled estimate of significance. To address the risk of false positives due to multiple comparisons across different dropout definitions and time points, we applied a Bonferroni correction to the pooled median *p*-values. All tests were conducted separately for each time point (baseline and follow-up), and for five operational definitions of dropout in a total of ten comparisons. Bonferroni-adjusted *p*-values were then used to determine statistical significance. The correction was applied by dividing the conventional alpha level (0.05) by the number of comparisons (10), yielding an adjusted significance threshold of *p* < 0.005.

To address RQs3 (usage patterns and association with dropout), we used K-means clustering to identify distinct usage patterns among participants based on their usage metrics (number of logins, total duration of logins, mean duration of logins, number of assigned chapters, number of requested modules, number of messages from therapist to patient, number of messages from patient to therapist, completion of assigned content). Instead of conducting survival analyses as preregistered, we relied on logistic regressions to examine the association between usage patterns and treatment dropout, with the different usage patterns (clusters identified in RQ3.1) as predictors. This change in analysis method was necessary due to our inability to identify a reliable time-to-event variable, as treatment dropout was only recorded at 6 months follow-up (t3) or had to be reported manually by the therapist. The results of this analysis are reported in detail in the supplement.

## Supplementary information

Supplement_Revision_formatted

## Data Availability

The dataset analyzed during the presented study is available in the OSF repository, 10.17605/OSF.IO/PRE87.

## Code availability

All code for the pre-processing and analysis is provided in the OSF repository 10.17605/OSF.IO/PRE87.

## References

[1] Ferrao Nunes-Z, K. et al. Blended psychological therapy for the treatment of psychological disorders in adult patients: systematic review and meta-analysis. *Interact. J. Med. Res* **13**, e49660 (2024). 39470720 10.2196/49660 PMC11558224

[2] Erbe, D. et al. Blending face-to-face and internet-based interventions for the treatment of mental disorders in adults: systematic review. *J. Med. Internet Res.* **19**, e306 (2017). 28916506 10.2196/jmir.6588 PMC5622288

[3] Kullgard, N., Holmqvist, R. & Andersson, G. Premature dropout from psychotherapy: prevalence, perceived reasons and consequences as rated by clinicians. *Clin. Psychol. Eur.* **4**, e6695 (2022). 36397946 10.32872/cpe.6695 PMC9667417

[4] Penix-Smith, E. A. et al. No client left behind: a meta-analysis of premature termination from psychotherapy in U.S. service members and veterans. *Am. Psychol.* **80**, 712–728 (2025). 38695780 10.1037/amp0001320

[5] Lewis, C. et al. Dropout from psychological therapies for post-traumatic stress disorder (PTSD) in adults: systematic review and meta-analysis. *Eur. J. Psychotraumatol.* **11**, 1709709 (2020). 32284816 10.1080/20008198.2019.1709709 PMC7144189

[6] Cooper, A. A. & Conklin, L. R. Dropout from individual psychotherapy for major depression: a meta-analysis of randomized clinical trials. *Clin. Psychol. Rev.* **40**, 57–65 (2015). 26067572 10.1016/j.cpr.2015.05.001

[7] Gersh, E. et al. Systematic review and meta-analysis of dropout rates in individual psychotherapy for generalized anxiety disorder. *J. Anxiety Disord.* **52**, 25–33 (2017). 29028610 10.1016/j.janxdis.2017.10.001

[8] Linardon, J. et al. Absolute and relative rates of treatment non-initiation, dropout, and attrition in internet-based and face-to-face cognitive-behavioral therapy: a meta-analysis of randomized controlled trials. *Cogn. Behav. Ther.* **4**, 1–14 (2025). 10.1080/16506073.2025.2542364 40757987

[9] Eysenbach, G. The law of attrition. *J. Med. Internet Res.* **7**, e11 (2005). 15829473 10.2196/jmir.7.1.e11 PMC1550631

[10] Forbes, A. et al. Assessing patient adherence to and engagement with digital interventions for depression in clinical trials: systematic literature review. *J. Med. Internet Res.* **25**, e43727 (2023). 37566447 10.2196/43727 PMC10457707

[11] Vöhringer, M. et al. Should I stay or must I go? Predictors of dropout in an internet-based psychotherapy programme for posttraumatic stress disorder in Arabic. *Eur. J. Psychotraumatol.* **11**, 1706297 (2020). 32082510 10.1080/20008198.2019.1706297 PMC7006804

[12] Karyotaki, E. et al. Predictors of treatment dropout in self-guided web-based interventions for depression: an ‘individual patient data’ meta-analysis. *Psychol. Med.* **45**, 2717–2726 (2015). 25881626 10.1017/S0033291715000665

[13] van Ballegooijen, W. et al. Adherence to Internet-based and face-to-face cognitive behavioural therapy for depression: a meta-analysis. *PLoS ONE* **9**, e100674 (2014). 25029507 10.1371/journal.pone.0100674 PMC4100736

[14] Børtveit, L. et al. Guided internet-delivered treatment for depression: scoping review. *JMIR Ment. Health* **9**, e37342 (2022). 36194467 10.2196/37342 PMC9579933

[15] Christensen, D. R. et al. Adding an internet-delivered treatment to an efficacious treatment package for opioid dependence. *J. Consult Clin. Psychol.* **82**, 964–972 (2014). 25090043 10.1037/a0037496 PMC4244262

[16] Campbell, A. N. et al. Internet-delivered treatment for substance abuse: a multisite randomized controlled trial. *Am. J. Psychiatry* **171**, 683–690 (2014). 24700332 10.1176/appi.ajp.2014.13081055 PMC4079279

[17] Kiluk, B. D. et al. Randomized trial of computerized cognitive behavioral therapy for alcohol use disorders: efficacy as a virtual stand-alone and treatment add-on compared with standard outpatient treatment. *Alcohol Clin. Exp. Res.* **40**, 1991–2000 (2016). 27488212 10.1111/acer.13162 PMC5008977

[18] Buelens, F. et al. Usage of unguided, guided, and blended care for depression offered in routine clinical care: Lessons learned. *Internet Inter.* **34**, 100670 (2023). 10.1016/j.invent.2023.100670 PMC10520335 37767005

[19] Cahill, J. et al. Outcomes of patients completing and not completing cognitive therapy for depression. *Br. J. Clin. Psychol.* **42**, 133–143 (2003). 12828803 10.1348/014466503321903553

[20] Moshe, I. et al. Digital interventions for the treatment of depression: a meta-analytic review. *Psychol. Bull.* **147**, 749–786 (2021). 34898233 10.1037/bul0000334

[21] Wright, J. H. et al. Computer-assisted cognitive-behavior therapy for depression: a systematic review and meta-analysis. *J. Clin. Psychiatry* **80**, 18r12188 (2019). 30900849 10.4088/JCP.18r12188

[22] Bremer, V. et al. Developing a process for the analysis of user journeys and the prediction of dropout in digital health interventions: machine learning approach. *J. Med. Internet Res.* **22**, e17738 (2020). 33112241 10.2196/17738 PMC7657718

[23] Moshe, I. et al. Predictors of dropout in a digital intervention for the prevention and treatment of depression in patients with chronic back pain: secondary analysis of two randomized controlled trials. *J. Med. Internet Res.* **24**, e38261 (2022). 36040780 10.2196/38261 PMC9472049

[24] Krijnen-de Bruin, E. et al. Usage intensity of a relapse prevention program and its relation to symptom severity in remitted patients with anxiety and depression: pre-post study. *JMIR Ment. Health* **9**, e25441 (2022). 35293876 10.2196/25441 PMC8968549

[25] Kemmeren, L. L. et al. Unraveling the black box: exploring usage patterns of a blended treatment for depression in a multicenter study. *JMIR Ment. Health* **6**, e12707 (2019). 31344670 10.2196/12707 PMC6686640

[26] Wu, M. S. et al. Predicting non-initiation of care and dropout in a blended care CBT intervention: Impact of early digital engagement, sociodemographic, and clinical factors. *Digit. Health* **8**, 20552076221133760 (2022). 36312847 10.1177/20552076221133760 PMC9608016

[27] Birnkammer, S. & Calvano, C. A creative and movement-based blended intervention for children in outpatient residential care: a mixed-method, multi-center, single-arm feasibility trial. *Children* **10**, 207 (2023). 36832336 10.3390/children10020207 PMC9954900

[28] Branquinho, M., Canavarro, M. C. & Fonseca, A. Blended CBT intervention vs. a guided web-based intervention for postpartum depression: results from a pilot randomized controlled trial. *Clin. Psychol. Psychother.* **31**, e70007 (2024). 39500299 10.1002/cpp.70007

[29] Nordby, E. S. et al. A blended intervention targeting emotion dysregulation in adults with attention-deficit/hyperactivity disorder: development and feasibility study. *JMIR Form. Res.* **8**, e53931 (2024). 38231536 10.2196/53931 PMC10831671

[30] Pérez, J. C. et al. An adjunctive internet-based intervention to enhance treatment for depression in adults: randomized controlled trial. *JMIR Ment. Health* **8**, e26814 (2021). 34927594 10.2196/26814 PMC8726028

[31] Breider, S. et al. Parent training for disruptive behaviors in referred children with autism spectrum disorder: a randomized controlled trial. *J. Autism Dev. Disord.* **56**, 481–498 (2026). 39331246 10.1007/s10803-024-06567-0 PMC12864344

[32] Forrer, F. et al. Binge-eating adolescent treatment (BEAT) - findings from a pilot study on effects and acceptance of a blended treatment program for youth with loss of control eating. *BMC Psychol.* **11**, 415 (2023). 38012794 10.1186/s40359-023-01429-3 PMC10683190

[33] Garety, P. et al. Effects of slowmo, a blended digital therapy targeting reasoning, on paranoia among people with psychosis: a randomized clinical trial. *JAMA Psychiatry* **78**, 714–725 (2021). 33825827 10.1001/jamapsychiatry.2021.0326 PMC8027943

[34] Kooistra, L. C. et al. Development and initial evaluation of blended cognitive behavioural treatment for major depression in routine specialized mental health care. *Internet Inter.* **4**, 61–71 (2016). 10.1016/j.invent.2016.01.003 PMC6096194 30135791

[35] Lundin, J. et al. Integrating digital and in-person therapy for PTSD: feasibility and acceptability of blended trauma-focused cognitive therapy in routine care. *Front. Psychiatry* **15**, 1447651 (2024). 39301223 10.3389/fpsyt.2024.1447651 PMC11410639

[36] Mathiasen, K. et al. The clinical effectiveness of blended cognitive behavioral therapy compared with face-to-face cognitive behavioral therapy for adult depression: randomized controlled noninferiority trial. *J. Med. Internet Res.* **24**, e36577 (2022). 36069798 10.2196/36577 PMC9543221

[37] Romijn, G. et al. Acceptability, effectiveness and cost-effectiveness of blended cognitive-behavioural therapy (bCBT) versus face-to-face CBT (ftfCBT) for anxiety disorders in specialised mental health care: a 15-week randomised controlled trial with 1-year follow-up. *PLoS ONE* **16**, e0259493 (2021). 34767575 10.1371/journal.pone.0259493 PMC8589191

[38] Schuster, R. et al. Effects, adherence, and therapists’ perceptions of web- and mobile-supported group therapy for depression: mixed-methods study. *J. Med. Internet Res.* **21**, e11860 (2019). 31066700 10.2196/11860 PMC6533044

[39] Fernández-Buendía, S. et al. A blended intervention for adjustment disorder: study protocol for a feasibility trial. *Internet Inter.* **35**, 100715 (2024). 10.1016/j.invent.2024.100715 PMC10837064 38313142

[40] Flückiger, C. et al. Assessing the alliance-outcome association adjusted for patient characteristics and treatment processes: a meta-analytic summary of direct comparisons. *J. Couns. Psychol.* **67**, 706–711 (2020). 32212755 10.1037/cou0000424 PMC7529648

[41] Musiat, P. et al. Impact of guidance on intervention adherence in computerised interventions for mental health problems: a meta-analysis. *Psychol. Med.* **52**, 229–240 (2022). 34802474 10.1017/S0033291721004621

[42] Baumeister, H. et al. The impact of guidance on Internet-based mental health interventions — A systematic review. *Internet Inter.* **1**, 205–215 (2014).

[43] Atik, E., Schückes, M. & Apolinário-Hagen, J. Patient and therapist expectations for a blended cognitive behavioral therapy program for depression: qualitative exploratory study. *JMIR Ment. Health* **9**, e36806 (2022). 36583934 10.2196/36806 PMC9840101

[44] Titzler, I. et al. Barriers and facilitators for the implementation of blended psychotherapy for depression: a qualitative pilot study of therapists’ perspective. *Internet Inter.* **12**, 150–164 (2018). 10.1016/j.invent.2018.01.002 PMC6096333 30135779

[45] Freidl, M. et al. Determinants of quality of life improvements in anxiety and depressive disorders-A longitudinal study of inpatient psychotherapy. *Front. Psychiatry* **13**, 937194 (2022). 36590609 10.3389/fpsyt.2022.937194 PMC9798124

[46] Murphy, S. T. et al. The therapeutic alliance and dropout in cognitive behavioral therapy of depression. *Psychother. Res.* **32**, 995–1002 (2022). 35041574 10.1080/10503307.2021.2025277

[47] Waumans, R. C. et al. Understanding and preventing nonadherence and treatment dropout in adolescents and young adults with anxiety and depressive disorders. *Front. Psychiatry* **14**, 1174285 (2023). 38076685 10.3389/fpsyt.2023.1174285 PMC10703356

[48] Karekla, M. et al. The phenomenon of treatment dropout, reasons and moderators in acceptance and commitment therapy and other active treatments: a meta-analytic review. *Clin. Psychol. Eur.* **1**, e2545 (2019).

[49] Alfonsson, S., Olsson, E. & Hursti, T. Motivation and treatment credibility predicts dropout, treatment adherence, and clinical outcomes in an internet-based cognitive behavioral relaxation program: a randomized controlled trial. *J. Med. Internet Res.* **18**, e52 (2016). 26957354 10.2196/jmir.5352 PMC4804106

[50] Wilhelmsen, M. et al. Motivation to persist with internet-based cognitive behavioural treatment using blended care: a qualitative study. *BMC Psychiatry* **13**, 296 (2013). 24199672 10.1186/1471-244X-13-296 PMC4226213

[51] Wells, S. Y. et al. Veterans’ reasons for dropping out of prolonged exposure therapy across three delivery modalities: a qualitative examination. *Psychol. Serv.* **20**, 483–495 (2023). 36326662 10.1037/ser0000714 PMC10154431

[52] Sabaté, E. Adherence to long-term therapies: evidence for action (*World Health Organization*, 2003).

[53] Donkin, L. et al. A systematic review of the impact of adherence on the effectiveness of e-therapies. *J. Med. Internet Res.* **13**, e52 (2011). 21821503 10.2196/jmir.1772 PMC3222162

[54] Sieverink, F., Kelders, S. M. & van Gemert-Pijnen, J. E. Clarifying the concept of adherence to ehealth technology: systematic review on when usage becomes adherence. *J. Med. Internet Res.* **19**, e402 (2017). 29212630 10.2196/jmir.8578 PMC5738543

[55] Barkham, M. et al. Dose-effect relations and responsive regulation of treatment duration: the good enough level. *J. Consult Clin. Psychol.* **74**, 160–167 (2006). 16551153 10.1037/0022-006X.74.1.160

[56] Chien, I. et al. A machine learning approach to understanding patterns of engagement with internet-delivered mental health interventions. *JAMA Netw. Open* **3**, e2010791 (2020). 32678450 10.1001/jamanetworkopen.2020.10791 PMC7368176

[57] Tricco, A. C. et al. PRISMA extension for scoping reviews (PRISMA-ScR): checklist and explanation. *Ann. Intern Med.* **169**, 467–473 (2018). 30178033 10.7326/M18-0850

[58] Munn, Z. et al. What are scoping reviews? Providing a formal definition of scoping reviews as a type of evidence synthesis. *JBI Evid. Synth.* **20**, 950–952 (2022). 35249995 10.11124/JBIES-21-00483

[59] Schaeuffele, C. et al. Increasing the effectiveness of psychotherapy in routine care through blended therapy with transdiagnostic online modules (PsyTOM): study protocol for a randomized controlled trial. *Trials* **23**, 830 (2022). 36180962 10.1186/s13063-022-06757-0 PMC9524091

[60] Schaeuffele, C. et al. Increasing the effectiveness of psychotherapy in routine care through transdiagnostic online modules? Randomized controlled trial investigating blended care. *J. Consult Clin. Psychol.* **94**, 11–25 (2026). 41538200 10.1037/ccp0000983

[61] Kroenke, K. et al. The PHQ-8 as a measure of current depression in the general population. *J. Affect Disord.* **114**, 163–173 (2009). 18752852 10.1016/j.jad.2008.06.026

[62] Kroenke, K. & Spitzer, R. The PHQ-9: a new depression diagnostic and severity measure. *Psychiatr. Ann.* **32**, 509–521 (2002).

[63] Gräfe, K. et al. Screening psychischer Störungen mit dem Gesundheitsfragebogen für Patienten (PHQ-D). *Diagnostica* **50**, 171–181 (2004).

[64] Löwe, B. et al. Validation and standardization of the generalized anxiety disorder screener (GAD-7) in the general population. *Med. Care* **46**, 266–274 (2008). 18388841 10.1097/MLR.0b013e318160d093

[65] Spitzer, R. L. et al. A brief measure for assessing generalized anxiety disorder: the GAD-7. *Arch. Intern Med.* **166**, 1092–1097 (2006). 16717171 10.1001/archinte.166.10.1092

[66] Diener, E. et al. The Satisfaction with life scale. *J. Pers. Assess.* **49**, 71–75 (1985). 16367493 10.1207/s15327752jpa4901_13

[67] Glaesmer, H. et al. The german version of the satisfaction with life scale (swls): psychometric properties, validity, and population-based norms. *Eur. J. Psychol. Assess.* **27**, 127–132 (2011).

[68] Schaeuffele, C. et al., Increasing the effectiveness of psychotherapy through transdiagnostic online modules? Randomized controlled trial comparing blended care to psychotherapy in routine care. 10.31234/osf.io/972z5 (2024). 10.1037/ccp0000983 41538200

[69] Eekhout, I., van de Wiel, M. A. & Heymans, M. W. Methods for significance testing of categorical covariates in logistic regression models after multiple imputation: power and applicability analysis. *BMC Med. Res. Methodol.* **17**, 129 (2017). 28830466 10.1186/s12874-017-0404-7 PMC5568368

