# Supplementary material for: Defining and reporting treatment dropout in blended therapy for mental health: scoping review and analysis
Source: NPJ Digit Med. 2026 Mar 17;9:245. doi: 10.1038/s41746-026-02546-0 (PMC13022307; doi:10.1038/s41746-026-02546-0)
Supplement: Supplementary file 1 — Supplement_Revision_formatted [file 41746_2026_2546_MOESM1_ESM.pdf]

Supplementary material.

Table of contents

Supplementary table 1. Overview of all treatment dropout definitions extracted from the literature…………………………………………….……..2

References. All included studies.……………………………………………………………………………………..……... 5

Supplementary table 2. Preferred Reporting Items for Systematic reviews and Meta-Analyses extension for Scoping Reviews (PRISMA-ScR) Checklist…….………………………………………………………………………………………..……...................................7

Supplementary table 3. Contents of digital modules in TONI.…………………………………………………………………...9

Supplementary figure 1. Elbow plot for cluster analysis………………………………………………………………………..10

Supplementary table 4. Logistic regression with treatment dropout according to different operational definitions as outcome and identified clusters for usage patterns as predictors……………………………………………………………………………………………………11

Supplementary table 1.

Overview of all treatment dropout definitions extracted from the literature.

**Authors**	**Definition extracted from publication**	**Quantification of digital component**	**Quantification of face-to-face component**	**Quantification: combination of digital and face-to-face component**	**Which component is part of the treatment dropout definition**	**Classification following Ferrao Nunes-Zlotkowski et al. (2024) :**<br>**1) integrated vs sequential**<br>**2) core vs supplementary**<br>**3) alternate vs case by case**<br>**4) personalized vs standardized**
Birnkammer and Calvano (2023)	usage of app until last module (or starting last module)	Yes (100%)	No	No	digital component	1) integrated<br>2)<br>3) alternate<br>4) standardized
Branquinho et al. (2024)	completion of at least 75% of all face-to-face sessions/phone calls	No	Yes (75%)	No	face-to-face component	1) integrated<br>2)<br>3) alternate<br>4) standardized
Breider et al. (2024)	categorized as "discontinued training" if not all digital and face-to-face modules were completed (100%)	No	No	Yes (100%)	Combination (additive)	1) integrated<br>2)<br>3) alternate<br>4) standardized
Fernández-Buendía et al. (2024)	participant will be considered a treatment dropout when, after meeting the eligibility criteria and signing the informed consent, they explicitly declare that they do not want to continue with treatment or when they have not responded to reminders.	No	No	No	Combination (not described)	1) integrated<br>2)<br>3)<br>4)
Forrer et al. (2023)	categorized as dropout if not completing all sessions (digital and face-to-face, 100%)	No	No	Yes (100%)	Combination (additive)	1) integrated<br>2)<br>3) alternate<br>4) standardized

Garety et al. (2021)	adherence was operationalized as at least 1 home screen interaction after a minimum of 3 therapy sessions and was recorded by system analytics	Yes (not described)	Yes (not described)	Yes (not described)	Combination (complex)	1) integrated<br>2)<br>3) alternate<br>4) standardized
Kooistra et al. (2016)	adherence was operationalized as completion of the face-to-face and digital sessions (100%)	Yes (100%)	Yes (100%)	Yes (100%)	Combination (additive)	1) integrated<br>2) f2f supplementary<br>3) alternate<br>4) standardized
Lundin et al. (2024)	Treatment dropout was defined as participants discontinuing, or completing less than 50% of the treatment (digital and face-to-face)	No	No	Yes (50%)	Combination (additive)	1) integrated<br>2)<br>3) alternate<br>4) standardized
Mathiasen et al. (2022)	categorized as dropout if completing less than 9 sessions (digital and face-to-face) out of 12 (75%)	No	No	Yes (75%)	Combination (additive)	1) integrated<br>2)<br>3) alternate<br>4) standardized
Nordby et al. (2024)	categorized as dropout if completing less than 6 group sessions (only face-to-face component) out of 8 (75%)	No	Yes (75%)	No	face-to-face component	1) integrated<br>2)<br>3) alternate<br>4) standardized

Pérez et al. (2021)	categorized as dropout if not completing all sessions (face-to-face, 100%)	No	Yes (not described)	No	face-to-face component	1) integrated<br>2)<br>3) alternate<br>4) standardized
Romijn et al. (2021)	completion was defined as finishing 15 sessions or dropping out due to remission (digital and face-to-face component, 100%)	No	No	Yes (100%)	Combination (additive)	1) integrated<br>2)<br>3) alternate<br>4) standardized
Schuster et al. (2019)	completion was defined as finishing the treatment with all blended therapy blocks	Yes (100%)	Yes (not described)	Yes (not described)	Combination (not described)	1) integrated<br>2)<br>3) alternate<br>4) standardized
Wu et al. (2022)	Categorized as dropout if patient stopped after a minimum of one face-to-face session (without describing the number of overall sessions)	No	Yes (not described)	No	face-to-face component	1) integrated<br>2)<br>3) alternate<br>4) personalized

References.

Birnkammer, S., & Calvano, C. (2023). A Creative and Movement-Based Blended Intervention for Children in Outpatient Residential Care: A Mixed-Method, Multi-Center, Single-Arm Feasibility Trial. *Children (Basel)*, *10*(2). https://doi.org/10.3390/children10020207

Branquinho, M., Canavarro, M. C., & Fonseca, A. (2024). Blended CBT Intervention vs. a Guided Web-Based Intervention for Postpartum Depression: Results From a Pilot Randomized Controlled Trial. *Clin Psychol Psychother*, *31*(6), e70007. https://doi.org/10.1002/cpp.70007

Breider, S., de Bildt, A., Greaves-Lord, K., Dietrich, A., Hoekstra, P. J., & van den Hoofdakker, B. J. (2024). Parent Training for Disruptive Behaviors in Referred Children with Autism Spectrum Disorder: A Randomized Controlled Trial. *J Autism Dev Disord*. https://doi.org/10.1007/s10803-024-06567-0

Fernández-Buendía, S., Cuijpers, P., Grimaldos, J., Díaz-García, A., Palau-Batet, M., & Quero, S. (2024). A blended intervention for adjustment disorder: Study protocol for a feasibility trial. *Internet Interv*, *35*, 100715. https://doi.org/10.1016/j.invent.2024.100715

Ferrao Nunes-Zlotkowski, K., Shepherd, H. L., Beatty, L., Butow, P., & Shaw, J. M. (2024). Blended Psychological Therapy for the Treatment of Psychological Disorders in Adult Patients: Systematic Review and Meta-Analysis. *Interact J Med Res*, *13*, e49660. https://doi.org/10.2196/49660

Forrer, F., Rubo, M., Meyer, A. H., & Munsch, S. (2023). Binge-eating adolescent treatment (BEAT) - findings from a pilot study on effects and acceptance of a blended treatment program for youth with loss of control eating. *BMC Psychol*, *11*(1), 415. https://doi.org/10.1186/s40359-023-01429-3

Garety, P., Ward, T., Emsley, R., Greenwood, K., Freeman, D., Fowler, D., Kuipers, E., Bebbington, P., Rus-Calafell, M., McGourty, A., Sacadura, C., Collett, N., James, K., & Hardy, A. (2021). Effects of SlowMo, a Blended Digital Therapy Targeting Reasoning, on Paranoia Among People With Psychosis: A Randomized Clinical Trial. *JAMA Psychiatry*, *78*(7), 714-725. https://doi.org/10.1001/jamapsychiatry.2021.0326

Kooistra, L. C., Ruwaard, J., Wiersma, J. E., van Oppen, P., van der Vaart, R., van Gemert-Pijnen, J., & Riper, H. (2016). Development and initial evaluation of blended cognitive behavioural treatment for major depression in routine specialized mental health care. *Internet Interv*, *4*, 61-71. https://doi.org/10.1016/j.invent.2016.01.003

Lundin, J., Jansson-Fröjmark, M., Gustafsson-Björverud, L., Grey, N., Santoft, F., Ehlers, A., Carlbring, P., Lundgren, T., Bragesjö, M., & Salomonsson, S. (2024). Integrating digital and in-person therapy for PTSD: feasibility and acceptability of blended trauma-focused cognitive therapy in routine care. *Front Psychiatry*, *15*, 1447651. https://doi.org/10.3389/fpsyt.2024.1447651

Mathiasen, K., Andersen, T. E., Lichtenstein, M. B., Ehlers, L. H., Riper, H., Kleiboer, A., & Roessler, K. K. (2022). The Clinical Effectiveness of Blended Cognitive Behavioral Therapy Compared With Face-to-Face Cognitive Behavioral Therapy for Adult Depression: Randomized Controlled Noninferiority Trial. *J Med Internet Res*, *24*(9), e36577. https://doi.org/10.2196/36577

Nordby, E. S., Guribye, F., Schønning, V., Andersen, S. L., Kuntsi, J., & Lundervold, A. J. (2024). A Blended Intervention Targeting Emotion Dysregulation in Adults With Attention-Deficit/Hyperactivity Disorder: Development and Feasibility Study. *JMIR Form Res*, *8*, e53931. https://doi.org/10.2196/53931

Pérez, J. C., Fernández, O., Cáceres, C., Carrasco Á, E., Moessner, M., Bauer, S., Espinosa-Duque, D., Gloger, S., & Krause, M. (2021). An Adjunctive Internet-Based Intervention to Enhance Treatment for Depression in Adults: Randomized Controlled Trial. *JMIR Ment Health*, *8*(12), e26814. https://doi.org/10.2196/26814

Romijn, G., Batelaan, N., Koning, J., van Balkom, A., de Leeuw, A., Benning, F., Hakkaart van Roijen, L., & Riper, H. (2021). Acceptability, effectiveness and cost-effectiveness of blended cognitive-behavioural therapy (bCBT) versus face-to-face CBT (ftfCBT) for anxiety disorders in specialised mental health care: A 15-week randomised controlled trial with 1-year follow-up. *PLoS One*, *16*(11), e0259493. https://doi.org/10.1371/journal.pone.0259493

Schuster, R., Kalthoff, I., Walther, A., Köhldorfer, L., Partinger, E., Berger, T., & Laireiter, A. R. (2019). Effects, Adherence, and Therapists' Perceptions of Web- and Mobile-Supported Group Therapy for Depression: Mixed-Methods Study. *J Med Internet Res*, *21*(5), e11860. https://doi.org/10.2196/11860

Wu, M. S., Chen, S. Y., Wickham, R. E., Leykin, Y., Varra, A., Chen, C., & Lungu, A. (2022). Predicting non-initiation of care and dropout in a blended care CBT intervention: Impact of early digital engagement, sociodemographic, and clinical factors. *Digit Health*, *8*, 20552076221133760. https://doi.org/10.1177/20552076221133760

Supplementary table 2.
Preferred Reporting Items for Systematic reviews and Meta-Analyses extension for Scoping Reviews (PRISMA-ScR) Checklist

SECTION	ITEM	PRISMA-ScR CHECKLIST ITEM	REPORTED ON PAGE #
**TITLE**			
Title	1	Identify the report as a scoping review.	#1
**ABSTRACT**			
Structured summary	2	Provide a structured summary that includes (as applicable): background, objectives, eligibility criteria, sources of evidence, charting methods, results, and conclusions that relate to the review questions and objectives.	#2
**INTRODUCTION**			
Rationale	3	Describe the rationale for the review in the context of what is already known. Explain why the review questions/objectives lend themselves to a scoping review approach.	#2-5
Objectives	4	Provide an explicit statement of the questions and objectives being addressed with reference to their key elements (e.g., population or participants, concepts, and context) or other relevant key elements used to conceptualize the review questions and/or objectives.	#5
**METHODS**			
Protocol and registration	5	Indicate whether a review protocol exists; state if and where it can be accessed (e.g., a Web address); and if available, provide registration information, including the registration number.	#17
Eligibility criteria	6	Specify characteristics of the sources of evidence used as eligibility criteria (e.g., years considered, language, and publication status), and provide a rationale.	#16
Information sources*	7	Describe all information sources in the search (e.g., databases with dates of coverage and contact with authors to identify additional sources), as well as the date the most recent search was executed.	#16
Search	8	Present the full electronic search strategy for at least 1 database, including any limits used, such that it could be repeated.	#16
Selection of sources of evidence†	9	State the process for selecting sources of evidence (i.e., screening and eligibility) included in the scoping review.	#16
Data charting process‡	10	Describe the methods of charting data from the included sources of evidence (e.g., calibrated forms or forms that have been tested by the team before their use, and whether data charting was done independently or in duplicate) and any processes for obtaining and confirming data from investigators.	#16
Data items	11	List and define all variables for which data were sought and any assumptions and simplifications made.	#16, #5-6
Critical appraisal of individual sources of evidence§	12	If done, provide a rationale for conducting a critical appraisal of included sources of evidence; describe the methods used and how this information was used in any data synthesis (if appropriate).	Not applicable
Synthesis of results	13	Describe the methods of handling and summarizing the data that were charted.	#5-6
**RESULTS**			
Selection of sources of evidence	14	Give numbers of sources of evidence screened, assessed for eligibility, and included in the review, with reasons	#5

SECTION	ITEM	PRISMA-ScR CHECKLIST ITEM	REPORTED ON PAGE #
		for exclusions at each stage, ideally using a flow diagram.	
Characteristics of sources of evidence	15	For each source of evidence, present characteristics for which data were charted and provide the citations.	S1
Critical appraisal within sources of evidence	16	If done, present data on critical appraisal of included sources of evidence (see item 12).	Not applicable
Results of individual sources of evidence	17	For each included source of evidence, present the relevant data that were charted that relate to the review questions and objectives.	#5-6, S1
Synthesis of results	18	Summarize and/or present the charting results as they relate to the review questions and objectives.	#6-7
**DISCUSSION**			
Summary of evidence	19	Summarize the main results (including an overview of concepts, themes, and types of evidence available), link to the review questions and objectives, and consider the relevance to key groups.	#9-10
Limitations	20	Discuss the limitations of the scoping review process.	#14-15
Conclusions	21	Provide a general interpretation of the results with respect to the review questions and objectives, as well as potential implications and/or next steps.	#15
**FUNDING**			
Funding	22	Describe sources of funding for the included sources of evidence, as well as sources of funding for the scoping review. Describe the role of the funders of the scoping review.	#19

JBI = Joanna Briggs Institute; PRISMA-ScR = Preferred Reporting Items for Systematic reviews and Meta-Analyses extension for Scoping Reviews.
* Where *sources of evidence* (see second footnote) are compiled from, such as bibliographic databases, social media platforms, and Web sites.
† A more inclusive/heterogeneous term used to account for the different types of evidence or data sources (e.g., quantitative and/or qualitative research, expert opinion, and policy documents) that may be eligible in a scoping review as opposed to only studies. This is not to be confused with *information sources* (see first footnote).
‡ The frameworks by Arksey and O'Malley (6) and Levac and colleagues (7) and the JBI guidance (4, 5) refer to the process of data extraction in a scoping review as data charting.
§ The process of systematically examining research evidence to assess its validity, results, and relevance before using it to inform a decision. This term is used for items 12 and 19 instead of "risk of bias" (which is more applicable to systematic reviews of interventions) to include and acknowledge the various sources of evidence that may be used in a scoping review (e.g., quantitative and/or qualitative research, expert opinion, and policy document).

From: Tricco AC, Lillie E, Zarin W, O'Brien KK, Colquhoun H, Levac D, et al. PRISMA Extension for Scoping Reviews (PRISMAScR): Checklist and Explanation. Ann Intern Med. 2018;169:467–473. doi: 10.7326/M18-0850.

Supplementary table 3.
Contents of digital modules in TONI.

Module	Description of major concept and component
Development	Chapter 1: Biopsychosocial model of distress; Chapter 2: Biographical history of symptoms; Chapter 3: Genogram and intergenerational mental health
Values and goals	Chapter 1: Motivation and barriers to change; Chapter 2: Identifying personal values; Chapter 3: Therapy goals
Mindfulness	Chapter 1: Introduction to mindfulness; Chapter 2: Mindfulness-based exercises (internal and external)
Getting active	Chapter 1: Relationship between activity and mood; Chapter 2: Identifying fulfilling and enjoyable activities; Chapter 3: Implementing activities in daily life
Thoughts	Chapter 1: Relationship between thoughts and emotions; Chapter 2: Life story shaping thoughts and beliefs; Chapter 3: Mentalization and different perspectives
Understanding feelings	Chapter 1: Understanding feelings and emotional stance; Chapter 2: Messages behind feelings; Chapter 3: Life story shaping emotional experience
Dealing with feelings	Chapter 1: Tolerating emotions; Chapter 2: Avoidance/defense mechanisms and new reactions
Self-worth and strengths	Chapter 1: Concept of self-worth and personal standards; Chapter 2: Kindness and self-compassion; Chapter 3: Identifying strengths and resources
Communication	Chapter 1: Meanings of messages; Chapter 2: Communication patterns and reflection of relationships; Chapter 3: Nonviolent communication of needs and wishes
Body and well-being	Chapter 1: Relationship between body and mind; Chapter 2: Eating and body image; Chapter 3: Sleep; Chapter 4: Movement; Chapter 5: Pain and bodily complaints; Chapter 6: Sexuality; Chapter 7: Stress
Addictive substances and behaviors	Chapter 1: Core concepts of substance and nonsubstance use/addictive behavior; Chapter 2: Pros and cons of substance use; Chapter 3: Dealing with cravings; Chapter 4: Relapse prevention and dealing with relapses
Collaboration	This module is targeted at a patient's significant other<br>Chapter 1: Significant other's own mental well-being; Chapter 2: Basics of therapy and emotional well-being; Chapter 3: Joint exercises for patients and significant others

Supplementary figure 1.
Elbow plot for cluster analysis.

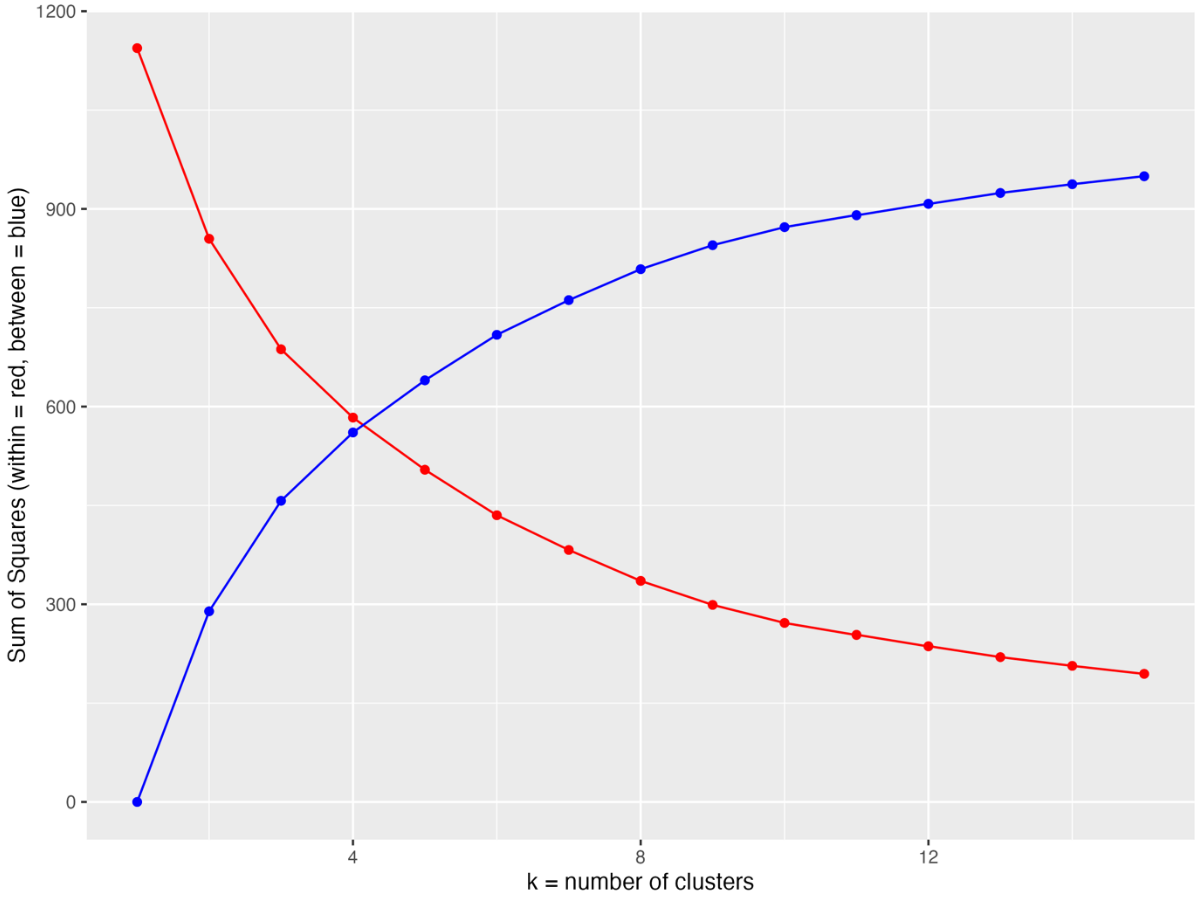

Silhouette scores
7 clusters: 0.29
6 clusters: 0.28
5 clusters: 0.26
4 clusters: 0.25
3 clusters: 0.24

While the elbow method suggests a solution around 4 clusters and the highest silhouette score was observed for 7 clusters (0.29), we selected a 5-cluster solution. This choice was informed by both statistical indicators and theoretical interpretability: the five-cluster solution yielded coherent and meaningful groupings that aligned best with the underlying structure of the data in the context of our research question. Although its silhouette score (0.26) was slightly lower than that for 6 or 7 clusters, it still reflects reasonable cluster separation. In contrast, the 6- and 7-cluster solutions, despite marginally higher silhouette scores, produced clusters that were difficult to interpret and did not offer added conceptual clarity.

Supplementary table 4.
Logistic regression with treatment dropout according to different operational definitions as outcome and identified clusters for usage patterns as predictors.

	OR	95% CI
**Definition 1 (therapist-only)**		
Cluster 1 (therapist contact)	Reference category	
Cluster 2 (selective usage)	5.00*	(1.03, 30.1)
Cluster 3 (minimal usage)	4.11*	(1.30, 14.08)
Cluster 4 (additional content)	6.33*	(1.62, 28.32)
Cluster 5 (moderate usage)	2.78	(0.86, 9.68)
**Definition 2 (digital-only)**		
Cluster 1 (therapist contact)	Reference category	
Cluster 2 (selective usage)	0.33	(0.01, 3.93)
Cluster 3 (minimal usage)	< .01	NA
Cluster 4 (additional content)	< .01	NA
Cluster 5 (moderate usage)	0.38	(0.02, 2.49)
**Definition 3 (100% digital & therapist)**		
Cluster 1 (therapist contact)	Reference category	
Cluster 2 (selective usage)	< .01	NA
Cluster 3 (minimal usage)	1.00	NA
Cluster 4 (additional content)	1.00	NA
Cluster 5 (moderate usage)	< .01	NA
**Definition 4 (75% digital & therapist)**		
Cluster 1 (therapist contact)	Reference category	
Cluster 2 (selective usage)	5.33	(0.38, 144.17)
Cluster 3 (minimal usage)	< .01	NA
Cluster 4 (additional content)	< .01	NA
Cluster 5 (moderate usage)	1.78	(0.20, 13.58)
**Definition 5 (50% digital & therapist)**		
Cluster 1 (therapist contact)	Reference category	
Cluster 2 (selective usage)	14*	(1.23, 385.0)
Cluster 3 (minimal usage)	< .01	NA
Cluster 4 (additional content)	34*	(3.25, 902.2)
Cluster 5 (moderate usage)	4	(0.47, 86.8)

The logistic regression suggested that clusters may be associated with treatment dropout, but results varied considerably across different dropout definitions (see Supplement). Statistically significant associations were observed for Definition 1 and 5. Here, Cluster 2 (selective usage) and 4 (additional content) showed higher dropout probabilities than Cluster 1 (therapist interaction). Definitions 2, 3 and 4 showed no significant effects, and exhibited signs of separation and instability, limiting interpretability for these results.
